# Effects of additive interactions among obesity, visceral adiposity, and sarcopenia on nonalcoholic fatty liver disease

**DOI:** 10.1038/s41598-023-30833-3

**Published:** 2023-03-03

**Authors:** Goh Eun Chung, Sohee Oh, Dong-Won Ahn, Su Hwan Kim, Yong Jin Jung, Ji Won Kim, Byeong Gwan Kim, Kook Lae Lee, Ji Bong Jeong

**Affiliations:** 1 Department of Internal Medicine and Healthcare Research Institute, Seoul National University Hospital Healthcare System Gangnam Center, Seoul, Republic of Korea; 2 Medical Research Collaborating Center, Seoul Metropolitan Government Seoul National University Boramae Medical Center, Seoul, Republic of Korea; 3 Department of Internal Medicine, Seoul National University College of Medicine, Seoul Metropolitan Government Seoul National University Boramae Medical Center, 20 Boramae-ro 5-gil, Dongjak-gu, Seoul, 07061 Republic of Korea; 4 Health Care Center, Seoul Metropolitan Government Seoul National University Boramae Medical Center, Seoul, Republic of Korea

**Keywords:** Hepatology, Endocrine system and metabolic diseases

## Abstract

Although the association of nonalcoholic fatty liver disease (NAFLD) with obesity or sarcopenia is known, few studies have investigated the combined effect of various body composition parameters on the risk of NAFLD. Thus, the aim of this study was to evaluate effects of interactions between various body composition parameters, including obesity, visceral adiposity, and sarcopenia, on NAFLD. Data of subjects who underwent health checkups between 2010 and December 2020 were retrospectively analyzed. Body composition parameters including appendicular skeletal muscle mass (ASM) and visceral adiposity were assessed using bioelectrical impedance analysis. Sarcopenia was defined as ASM/weight beyond two standard deviations below the gender-specific mean for healthy young adults. NAFLD was diagnosed using hepatic ultrasonography. Interaction analyses, including relative excess risk due to interaction (RERI), synergy index (SI), and attributable proportion due to interaction (AP), were performed. Among a total of 17,540 subjects (mean age: 46.7 years, 49.4% males), the prevalence of NAFLD was 35.9%. The odds ratio (OR) of interaction between obesity and visceral adiposity affecting NAFLD was 9.14 (95% CI: 8.29–10.07). The RERI was 2.63 (95% CI: 1.71–3.55), SI was 1.48 (95% CI: 1.29–1.69) and AP was 29%. The OR of interaction between obesity and sarcopenia affecting NAFLD was 8.46 (95% CI: 7.01–10.21). The RERI was 2.21 (95% CI: 0.51–3.90). SI was 1.42(95% CI: 1.11–1.82) and AP was 26%. The OR of interaction between sarcopenia and visceral adiposity affecting NAFLD was 7.25 (95% CI: 6.04–8.71), however, there was no significant additive interaction with RERI = 0.87 (95% CI: −0.76 to 2.51). Obesity, visceral adiposity, and sarcopenia were found to be positively associated with NAFLD. Obesity, visceral adiposity, and sarcopenia were found to have additive interaction effects on NAFLD.

## Introduction

Nonalcoholic fatty liver disease (NAFLD) has been recognized as the leading cause of chronic liver disease, with prevalence up to 20–30% in the general population^1^. Although NAFLD mostly follows a benign clinical course, some patients might experience progression of liver disease^2,3^. In addition, patients with nonalcoholic steatohepatitis have been found to have a higher liver-related mortality than the general population^4^. Since NAFLD is closely associated with various metabolic conditions such as obesity, type 2 diabetes, and dyslipidemia^5^, it might be accompanied by changes in body composition.

Sarcopenia is characterized as an age-related progressive loss of skeletal muscle with low muscle strength with/without physical performance^6^. It has been associated with increases of metabolic diseases, cardiovascular diseases, and mortality^7–9^. Growing evidence has shown significant associations between sarcopenia and NAFLD^10,11^. Both sarcopenia and NAFLD share the same primary pathophysiology as insulin resistance^12,13^. A recent review article has reported an interaction between sarcopenia and NAFLD demonstrating shared common mechanisms and the bidirectional relationship^14^. However, no previous study investigating the interactive effects of sarcopenia and NAFLD have been found.

Since the association of NAFLD with obesity or skeletal muscle is known, the coexistence of obesity, visceral adiposity, sarcopenia could potentiate each other and maximize their adverse effects on NAFLD. Therefore, we aimed to quantify additive interactive effects of various body composition parameters on the risk of NAFLD.

## Materials and methods

### Study population

**Figure 1:**
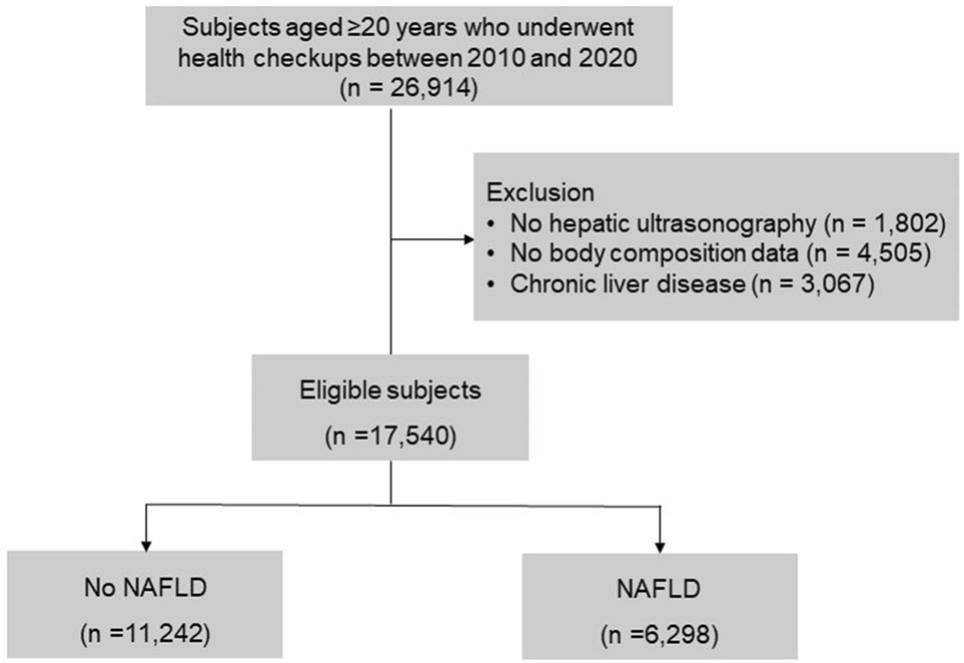
Flow chart.

In this retrospective study, subjects who received voluntary routine health checkups at Boramae Health Care Center between 2010 and 2020 were consecutively enrolled. Most of them had no symptoms and voluntarily underwent heath examinations. In the case of subjects who underwent twice or more exams, results of the primary test were used. Initially, a total of 26,914 adults aged ≥ 20 years were enrolled. Among them, 1802 subjects with no hepatic ultrasonography data and 4505 subjects with no body composition analysis were excluded. We also excluded subjects who had potential cause of chronic liver disease, including 806 who were positive for the hepatitis B virus or positive for the hepatitis C virus, 2258 who had a significant alcohol intake (> 210 g/week for males and > 140 g/week for females)^15^, and three who had liver cancer. Finally, 17,540 subjects were included for the analysis (Fig. 1).

This study was approved by the Institutional Review Board (IRB) of Boramae Medical Center (No. 30-2022-93). The requirement for written informed consent was waived by IRB of Boramae Medical Center due to the retrospective nature of this study. This study was conducted in accordance with the Declaration of Helsinki***.***

### Clinical assessments

Each subject in this study completed a questionnaire regarding their past medical history. Current smokers were defined as those who smoked at least one cigarette per day for the past 12 months. Laboratory tests were performed after an overnight 8-h fasting including serum aspartate aminotransferase (AST), alanine aminotransferase (ALT), total cholesterol, triglycerides (TG), high-density lipoprotein cholesterol (HDL-C), low-density lipoprotein cholesterol (LDL-C), glucose, hemoglobin A1c (HbA1c); and C-reactive protein (CRP).

### Anthropometric assessments

On the day of the health examination, participants received an anthropometric assessment. Body mass index (BMI) was calculated as follows: weight (kg) divided by square of height (m^2^). Waist circumference (WC) was measured at the umbilicus level with participants in a standing position. Systolic and diastolic blood pressure were measured twice with mean values reported.

Body composition analysis was performed by an experienced nurse using Inbody 720 (Biospace Co., Seoul, Korea) according to the manufacturer’s protocol^16^. Subjects were instructed to grasp handles of the analyzer in a standing position for 5–10 min with legs slightly apart and arms slightly abducted from the trunk. Once the measurements became stabilized, the analyzer performed multi-frequency measurements and provided impedances to each segment, including the trunk and extremities, to estimate the appendicular skeletal muscle mass (ASM). Skeletal muscle mass and visceral fat area (VFA) were automatically calculated. ASM and VFA measurements using the Inbody 720 were reported to have a high correlation with the results using computed tomography (CT)^17^. Among the study population, 1,927 subjects underwent CT examination. Pearson’s bivariate correlation analysis showed a significant strong correlation between measurements with Inbody 720 and those measured with CT (ASM, r = 0.898, *P* < 0.001 and VFA, r = 0.714, *P* < 0.001) (Supplementary Figs. S1, S2).

### Definitions

The presence of diabetes mellitus (DM) was defined as fasting serum glucose ≥ 126 mg/dL, HbA1c level ≥ 6.5%, or use of anti-diabetic medications. The presence of hypertension (HT) was defined as a systolic blood pressure ≥ 140 mmHg, a diastolic blood pressure ≥ 90 mmHg, or use of antihypertensive medications. The presence of dyslipidemia (DL) was defined as the serum levels of TG ≥ 150 mg/dL, HDL-C < 40 mg/dL in males and < 50 mg/dL in females, or the use of lipid-lowering medications.

To define obesity, we used a cut-off level of BMI ≥ 25 kg/m^2^ based on the criteria for the Asia–Pacific region. To assess visceral adiposity, VFA was used, and subjects with VFA ≥ 100 cm^2^ were placed in the visceral adiposity group^18^. ASM was calculated as the sum of the lean muscle mass of both upper and lower extremities. Sarcopenia was defined as ASM% (calculated as, ASM × 100/Weight) beyond two standard deviations below the gender-specific mean for healthy young adults (ASM% < 29.0 in males and < 22.9 in females) based on the Korean population data^19^.

### Measurement of NAFLD

Hepatic ultrasonography (Philips iU22; Philips Healthcare, Amsterdam, The Netherlands) was performed to diagnose fatty liver by experienced radiologists who were unaware to the details of subjects. The diagnosis of fatty liver was made on the ultrasonographic findings consistent with ‘‘bright liver’’ and a sharp contrast between hepatic and renal parenchyma, focal sparing, vessel blurring, and narrowing of the hepatic venous lumen^20,21^. We defined advanced fibrosis as high probability of fibrosis with NAFLD fibrosis score (NFS) > 0.67^22^.

### Statistical analysis

Continuous variables were expressed as mean ± SD or median (IQR) for according to the normality assumption. Categorical variables were expressed as numbers and percentages. Comparisons of continuous variables between groups were performed using Student’s *t*-test or Mann–Whitney U test. Categorical variables were compared using either the chi-square test or Fisher’s exact test. We performed a logistic regression analysis to estimate the risk of NAFLD with odds ratios (ORs) calculated. In the multivariate regression model, we adjusted age, sex, HT, DM, DL, smoking and CRP level.

To calculate the additive interaction effect by cross-analysis, an additive model was used. We calculated the relative excess risk due to interaction (RERI), synergy index (SI), and attributable proportion due to interaction (AP) as follows^23,24^:

$$\begin{gathered} RERI \, = \, RR_{11} - \, RR_{10} - \, RR_{01} + \, 1 \hfill \\ SI = \, \left( {RR_{11} - \, 1} \right)/(\left( {RR_{01} - \, 1} \right) \, + \, \left( {RR_{10} - \, 1} \right)) \hfill \\ AP \, = \, (RR_{11} - \, RR_{10} - \, RR_{01} + \, 1)/RR_{11} = \, RERI/RR_{11} \hfill \\ \end{gathered}$$

where RR_ab_ is the relative risk (RR) in the group with exposures a and b (1 = exposed, 0 = not exposed) compared to the non-double exposed group.

An RERI or AP of 0 means no additive interaction and RERI or AP ≥ 0 indicates a positive interaction^25^. An interaction was considered significant if the 95% confidence interval (CI) of SI did not contain 1 and the 95% CI of RERI and AP did not contain 0^26^.

## Results

### Clinical characteristics according to the presence of NAFLD

Among the total of 17,540 subjects, the mean age was 46.7 ± 13.4 years. There were 49.4% males. Baseline characteristics of the study population according to the presence of NAFLD are summarized in Table 1. Individuals with NAFLD were older. They had higher percentage of males. They also had higher BMI, WC, and VFA than those without NALFD (all *P* < 0.001). In addition, individuals with NAFLD had higher prevalence of DM, HT, and DL. They were more likely to be current smokers with higher blood pressure and higher serum levels of AST, ALT, total cholesterol, triglycerides, LDL-C, fasting glucose, and HbA1c but lower levels of HDL-cholesterol than those without NAFLD (all *P* < 0.001).

**Table 1:** Comparison of baseline characteristics according to the presence of NAFLD.

	No NAFLD (N = 11,242)	NAFLD (N = 6298)	*P*
Age (years)	45.5 ± 13.9	48.8 ± 12.2	< 0.001
Male,* n* (%)	4618 (41.1)	4054 (64.4)	< 0.001
Height (cm)	164.5 ± 8.7	166.7 ± 9.2	< 0.001
Weight (kg)	60.0 ± 10.2	70.9 ± 11.2	< 0.001
BMI (kg/m^2^)	22.1 ± 2.6	25.4 ± 2.6	< 0.001
Obesity (BMI ≥ 25 kg/m^2^),* n* (%)	1555 (13.8)	3470 (55.1)	< 0.001
WC (cm)	76.5 ± 8.5	86.0 ± 7.8	< 0.001
Visceral fat area (cm^2^)	71.9 [55.2; 90.9]	104.3 [86.7; 123.7]	< 0.001
Visceral adiposity,* n* (%)	1849 (16.4)	3528 (56.0)	< 0.001
ASM (kg)	17.2 [14.8; 21.9]	21.6 [16.9; 24.6]	< 0.001
ASM%, total	30.2 [27.6; 33.1]	30.0 [26.7; 32.0]	< 0.001
Male	33.5 [31.9; 35.2]	31.5 [30.1; 32.8]	< 0.001
Female	28.0 [26.3; 29.8]	25.5 [24.2; 27.0]	< 0.001
Sarcopenia, *n* (%)	261 (2.3)	694 (11.0)	< 0.001
SBP (mmHg)	113.2 ± 14.8	122.0 ± 14.6	< 0.001
DBP (mmHg)	76.7 ± 10.4	82.2 ± 10.6	< 0.001
AST (IU/L)	22 [18; 27]	25 [21; 32]	< 0.001
ALT (IU/L)	17 [13; 24]	28 [20; 42]	< 0.001
Cholesterol (mg/dL)	187 [166; 210]	199 [176; 224]	< 0.001
TG (mg/dL)	72 [55; 99]	119 [85; 168]	< 0.001
HDL-c (mg/dL)	58 [49; 68]	47 [41; 55]	< 0.001
LDL-c (mg/dL)	110 [90; 133]	123 [100; 146]	< 0.001
Fasting glucose (mg/dL)	88 [82; 94]	94 [88; 103]	< 0.001
Hb A1c (%)	5.4 [5.2; 5.6]	5.6 [5.4; 6.0]	< 0.001
CRP (mg/dL)	0.04 [0.02; 0.09]	0.09 [0.04; 0.17]	< 0.001
Diabetes mellitus, *n* (%)	490 (4.4)	964 (15.3)	< 0.001
Hypertension, *n* (%)	2287 (20.3)	2569 (40.8)	< 0.001
Dyslipidemia, *n* (%)	2223 (19.8)	3272 (52.0)	< 0.001
Smoking, *n* (%)			< 0.001
Never	8043 (71.5)	3538 (56.2)	
Former	1546 (13.8)	1328 (21.1)	
Current	1653 (14.7)	1432 (22.7)	

Data are shown as the mean ± SD, median [interquartile range, IQR] or *n* (%).

*NAFLD* nonalcoholic fatty liver disease, *BMI* body mass index, *WC* waist circumference, *ASM* appendicular muscle mass, *SBP* systolic blood pressure, *DBP* diastolic blood pressure, *AST* aspartate aminotransferase, *ALT* alanine aminotransferase, *TG* triglycerides, *HDL* high-density lipoprotein, *LDL* low-density lipoprotein, *HbA1C* hemoglobin A1 C, *CRP* C-reactive protein.

### Body composition and NAFLD

To evaluate associations of NAFLD with obesity, visceral adiposity, and sarcopenia, a logistic analysis was performed for the risk for NAFLD. When a multivariable logistic regression analysis was performed after adjusting for age, sex, HT, DM, DL, smoking, and CRP, NAFLD showed significant associations with obesity, visceral adiposity, and sarcopenia [obesity, OR (odds ratio): 5.53, 95% confidence interval (CI): (5.11–5.97); visceral adiposity, OR: 4.37, 95% CI: 4.05–4.72; and sarcopenia, OR: 3.53 95% CI: 3.01–4.14, Table 2].

**Table 2:** Multivariable analyses of the risk for NAFLD.

	Obesity (BMI ≥ 25 kg/m^2^)			Visceral adiposity			Sarcopenia		
	OR	95% CI	*P*-value	OR	95% CI	*P*-value	OR	95% CI	*P*-value
Crude	7.64	7.11–8.22	< 0.001	6.47	6.03–6.94	< 0.001	5.21	4.50–6.03	< 0.001
Multivariate*	5.53	5.11–5.97	< 0.001	4.37	4.05–4.72	< 0.001	3.53	3.01–4.14	< 0.001

*NAFLD* nonalcoholic fatty liver disease, *OR* odds ratio, *CI* confidence interval.

*Adjusted for age, sex, hypertension, diabetes mellitus, dyslipidemia, smoking and C-reactive protein levels.

### Interaction effect of body composition on NAFLD

**Figure 2:**
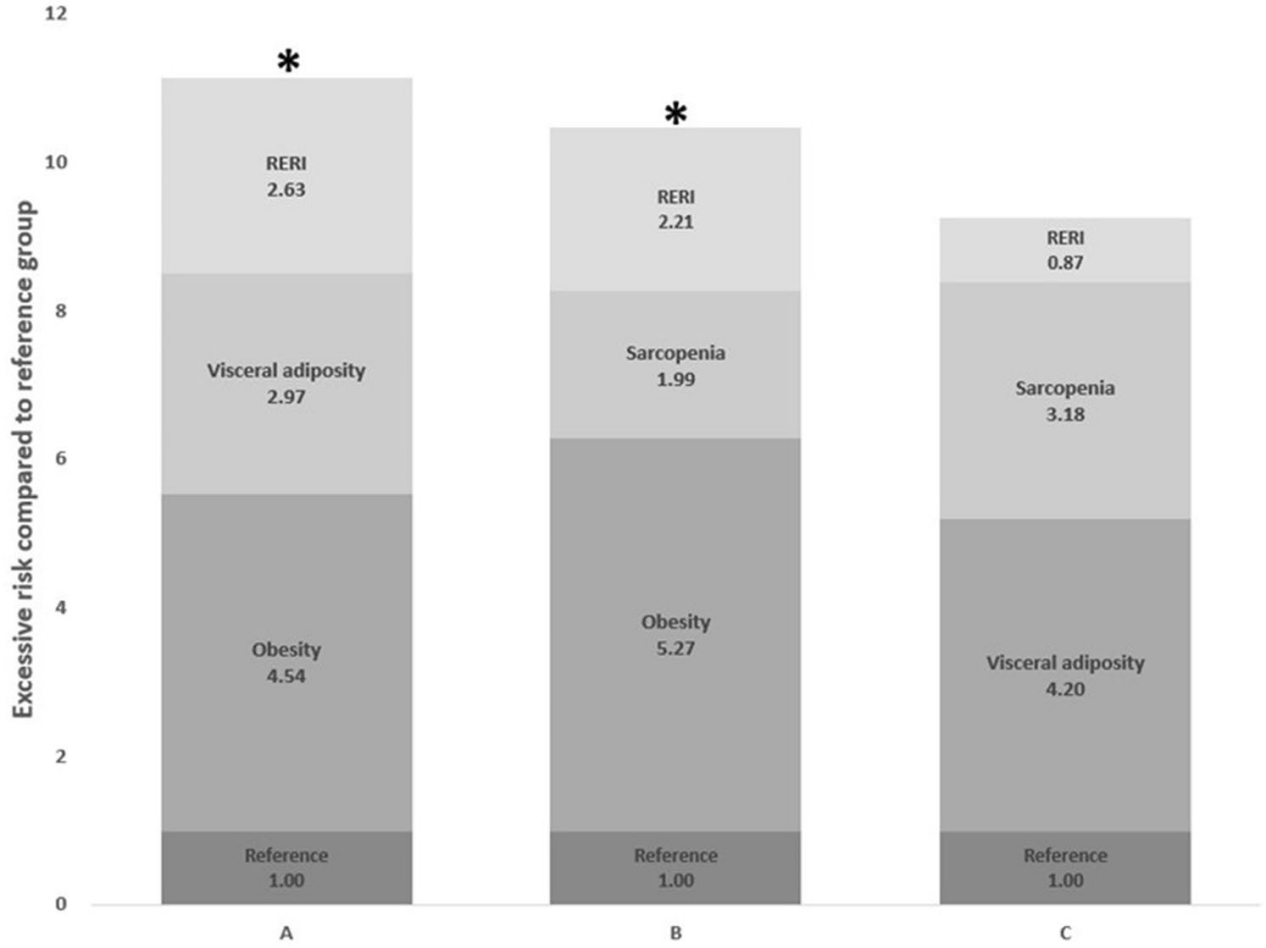
Additive interaction effect among obesity, visceral adiposity, sarcopenia on the presence of NAFLD. (**A**) Additive interaction between obesity and visceral adiposity. (**B**) Additive interaction between obesity and sarcopenia. (**C**) Additive interaction between visceral adiposity. Reference group: without obesity, visceral adiposity, or sarcopenia. *RERI* the relative excess risk due to the interaction. Asterisk: statistical significance.

To explore effects of interactions between obesity, visceral adiposity and sarcopenia on the risk of NAFLD, interactive effect analysis was performed. Figure 2A shows results of interactive effects between obesity and visceral adiposity on NAFLD. The OR of interaction between obesity and visceral adiposity was 9.14 (95% CI, 8.29–10.07). When we assessed the additive interaction effect, positive RERI, AP, and SI were observed between obesity and visceral adiposity related to NAFLD: RERI = 2.63 (95% CI, 1.71–3.55) and SI = 1.48 (95% CI, 1.29–1.69). By calculating AP, we found that 29% of the risk of NAFLD was due to an additive interaction between obesity and visceral adiposity (Table 3).

**Table 3:** The additive interaction between obesity and visceral adiposity on the risk of NAFLD.

Obesity	Visceral adiposity	Number of NAFLD	Number of no-NAFLD	OR (95% CI)
–	–	1902	8700	1
+	–	868	693	4.54 (4.04–5.10)
–	+	926	987	2.97 (2.66–3.32)
+	+	2602	862	9.14 (8.29–10.07)

RERI (95% CI) = 2.63 (1.71–3.55), SI (95% CI) = 1.48 (1.29–1.69), AP (95% CI) = 28.78 (20.47–37.08).

OR are adjusted for age, sex, hypertension, diabetes mellitus, dyslipidemia, smoking and C-reactive protein.

*NAFLD* nonalcoholic fatty liver disease, *OR* odds ratio, *CI* confidence interval, *RERI* relative excess risk due to interaction, *SI* synergy index, *AP* attributable proportion due to interaction.

When we evaluated the interaction effect between obesity and sarcopenia on NAFLD, the OR of interaction was 8.46 (95% CI, 7.01–10.21). RERI was larger than 0, 2.21 (95% CI, 0.51–3.90) and SI was 1.42 (95% CI, 1.11–1.82) (Fig. 2B, Table 4). The result of AP indicated that 26% of the risk of NAFLD was attributable to the interaction between obesity and sarcopenia. On the contrary there was no significantly additive interaction effect between sarcopenia and visceral adiposity on the risk of NAFLD (RERI = 0.87 [95% CI: −0.76 to 2.51]; SI = 1.16 [95% CI: 0.88–1.54]) (Fig. 2C, Table 5).

**Table 4:** The additive interaction between obesity and sarcopenia on the risk of NAFLD.

Obesity	Sarcopenia	Number of NAFLD	Number of no-NAFLD	OR (95% CI)
−	−	2749	9587	1
+	−	2855	1394	5.27 (4.85–5.72)
−	+	79	100	1.99 (1.44–2.75)
+	+	615	161	8.46 (7.01–10.21)

RERI (95% CI) = 2.21 (0.51–3.90), SI (95% CI) = 1.42 (1.11–1.82), AP (95% CI) = 26.06 (10.16–41.95).

OR are adjusted for age, sex, hypertension, diabetes mellitus, dyslipidemia, smoking and C-reactive protein.

*NAFLD* nonalcoholic fatty liver disease, *OR* odds ratio, *CI* confidence interval, *RERI* relative excess risk due to interaction, *SI* synergy index, *AP* attributable proportion due to interaction.

**Table 5:** The additive interaction between visceral adiposity and sarcopenia on the risk of NAFLD.

Visceral adiposity	Sarcopenia	Number of NAFLD	Number of no-NAFLD	OR (95% CI)
−	−	2674	9311	1
+	−	2930	1670	4.20 (3.87–4.55)
−	+	96	82	3.18 (2.31–4.37)
+	+	598	179	7.25 (6.04–8.71)

RERI (95% CI) = 0.87 (−0.76 to 2.51), SI (95% CI) = 1.16 (0.88–1.54), AP (95% CI) = 12.06 (−8.90 to 33.03).

OR are adjusted for age, sex, hypertension, diabetes mellitus, dyslipidemia, smoking and C-reactive protein.

*NAFLD* nonalcoholic fatty liver disease, *OR* odds ratio, *CI* confidence interval, *RERI* relative excess risk due to interaction, *SI* synergy index, *AP* attributable proportion due to interaction.

Next, we assessed liver fibrosis using NFS in patients with NAFLD. As a result, there was a significant association between obesity, visceral adiposity, sarcopenia and advanced fibrosis (Table 6). When we evaluated additive interactive effects among body composition parameters for advanced fibrosis, there was no significant additive interactions between obesity and visceral adiposity [RERI (95% CI), 0.12 (−0.29 to 0.52)], obesity and sarcopenia (RERI, −0.18), visceral adiposity and sarcopenia (RERI, −0.15), respectively.

**Table 6:** Multivariable analyses of the risk for advanced fibrosis in patients with NAFLD.

	Obesity (BMI ≥ 25 kg/m^2^)			Visceral adiposity			Sarcopenia		
	OR	95% CI	*P*-value	OR	95% CI	*P*-value	OR	95% CI	*P*-value
Crude	1.42	1.26–1.60	< 0.001	1.36	1.20–1.53	< 0.001	1.65	1.39–1.97	< 0.001
Multivariate*	1.72	1.49–1.98	< 0.001	1.23	1.07–1.42	0.005	1.32	1.07–1.61	0.009

*NAFLD* nonalcoholic fatty liver disease, *OR* odds ratio, *CI* confidence interval.

*Adjusted for age ≥ 50 years, sex, hypertension, diabetes mellitus, dyslipidemia, smoking and C-reactive protein levels.

## Discussion

In the current study, we demonstrated effects of additive interactions between obesity, visceral adiposity, and sarcopenia on the risk of NAFLD even after adjusting for multiple possible confounders. To the best of our knowledge, this is the first study to quantify additive interactive effects of adverse body composition parameters including obesity, visceral adiposity, and sarcopenia on the risk of NAFLD. quantified the interaction effect between body composition parameters on NAFLD by applying additive models and found that body composition parameters had positive interactions with NAFLD.

The close link between body composition parameters and NAFLD has been investigated. Previous studies have shown that obesity^27,28^, visceral adiposity^29,30^, and sarcopenia^13,31^, are associated with an increased risk for NAFLD. Obesity seems to play a role in both the initial process of simple hepatic steatosis and its progression to advanced liver disease^32^. Excess of free fatty acids derived from visceral adipose tissue (VAT) is considered as an important factor contributing to liver injury in NAFLD^33^. In addition, adipokines secreted from VAT and lipid accumulation in the liver can promote inflammation in the liver^33^. Regarding sarcopenia, since skeletal muscle is the primary tissue responsible for insulin-mediated glucose disposal^34,35^, insulin resistance, which is the main pathology of NAFLD, might have a common role in skeletal muscle and liver. The accumulation of visceral adiposity is the main link to sarcopenia in NAFLD through myosteatosis^36^. The coexistence of sarcopenia and visceral fat, which may attribute to the muscle-liver-adipose tissue axis in patients with NAFLD^13^.

However, most of these studies only analyzed the association of a single body composition parameter as a risk factor with NAFLD. Studies analyzing the association of multiple body composition parameters with NAFLD focusing on their interactive effects on the risk of NAFLD have been rare. Since interaction on the additive scale is more likely to reflect biological interactions, the interaction measurement between body composition parameters enables the estimation of an excess risk of NAFLD due to both exposures. If the interaction is greater than the sum of two risk factors, it means that there is an additive interaction effect^37^.

In this study, we quantified the interaction effect between body composition parameters on NAFLD by applying additive models and found that body composition parameters had positive interactions with NAFLD. RERI is the excess risk due to the interaction relative to the risk without exposure. It is used for quantitative analysis of interaction. AP refers to the attributable proportion of disease that is due to the interaction among individuals with both exposures. Thus, AP is used to calculate the proportion attributable to interaction after background effects are removed. This study showed that obesity and visceral adiposity have additive interaction with NAFLD, and the AP was 29%. It indicates that 29% of NAFLD was attributable to the interaction of them, when exposed to both obesity and visceral adiposity. Similarly, the AP between obesity and sarcopenia was 26%, indicating that 26% of NAFLD was attributable to the interaction of them, when exposed to both obesity and sarcopenia.

In this study, there were significant additive interactive effects of obesity and visceral adiposity, obesity and sarcopenia on NAFLD, but visceral adiposity and sarcopenia was not. These results suggest an independent rather than additive interactive effect of visceral adiposity and sarcopenia on NAFLD. The mechanism for these findings is unclear, but a possible explanation may relate to negative multiplicative interaction between visceral adiposity and sarcopenia (OR: 0.54, 95% CI: 0.37–0.78, *P* = 0.001, by multivariable binary logistic regression using the multiplicative term [visceral adiposity*sarcopenia]) (data not shown). However, further research is needed on this issue. Since body composition parameters including obesity, visceral adiposity, and skeletal muscles are modifiable factors, an effective intervention program such as diet control and regular physical activity could help reduce the risk of NAFLD in individuals with obesity, visceral adiposity, and/or sarcopenia.

This study also has several limitations. First, we determined the skeletal muscle mass using the bioimpedance analysis (BIA) method instead of CT or dual-energy X-ray absorptiometry. However, CT has limitations such as radiation risk and high cost. Although results might be influenced by body water component, BIA is convenient as it is a non-invasive, easily applicable, and inexpensive method to assess body composition^38^. It is also validated for multiethnic adults^39,40^. In addition, BIA-based sarcopenia cannot be linked to symptoms but, clinicians can advise effective lifestyle modification programs to reduce the risk of NAFLD for people measuring muscle mass for purposes such as health screenings. Second, functional aspects of skeletal muscle (such as muscle strength or physical performance), which are components for diagnostic criteria of sarcopenia, could not evaluated in this study. Third, although liver biopsy is the gold standard for the diagnosis and grading of NAFLD, we were unable to obtain liver histological samples because its invasiveness for asymptomatic subjects who underwent health check-ups. In addition, ultrasonography is a non-invasive and widely available method for detecting of hepatic steatosis, but it may produce false-negative results when fatty infiltration of the liver is less than 30%^41^.

In summary, obesity, visceral adiposity, and sarcopenia were found to be positively associated with NAFLD. In addition, obesity, visceral adiposity, and sarcopenia were showed interaction effects on NAFLD on the additive scale.

## Supplementary Information

Supplementary Figures.

## Data Availability

The datasets used and/or analyzed during the current study are available from the corresponding author on reasonable request.
