# Supplementary material for: Effects of additive interactions among obesity, visceral adiposity, and sarcopenia on nonalcoholic fatty liver disease
Source: Sci Rep. 2023 Mar 3;13:3628. doi: 10.1038/s41598-023-30833-3 (PMC9984466; doi:10.1038/s41598-023-30833-3)

Supplementary Figure S1. Correlation between ASM measured by Inbody 720 and ASM measured by computed tomography.

ASM, appendicular skeletal muscle mass; CT, computed tomography

Supplementary Figure S2. Correlation between VFA measured by Inbody 720 and VFA measured by computed tomography.

VFA, visceral fat area; CT, computed tomography


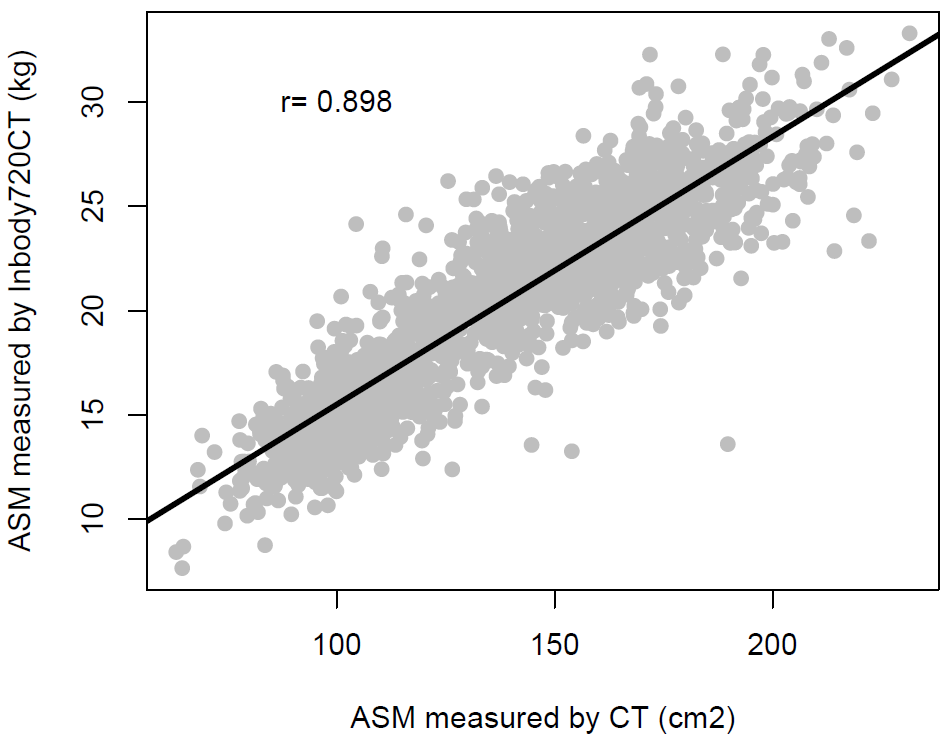


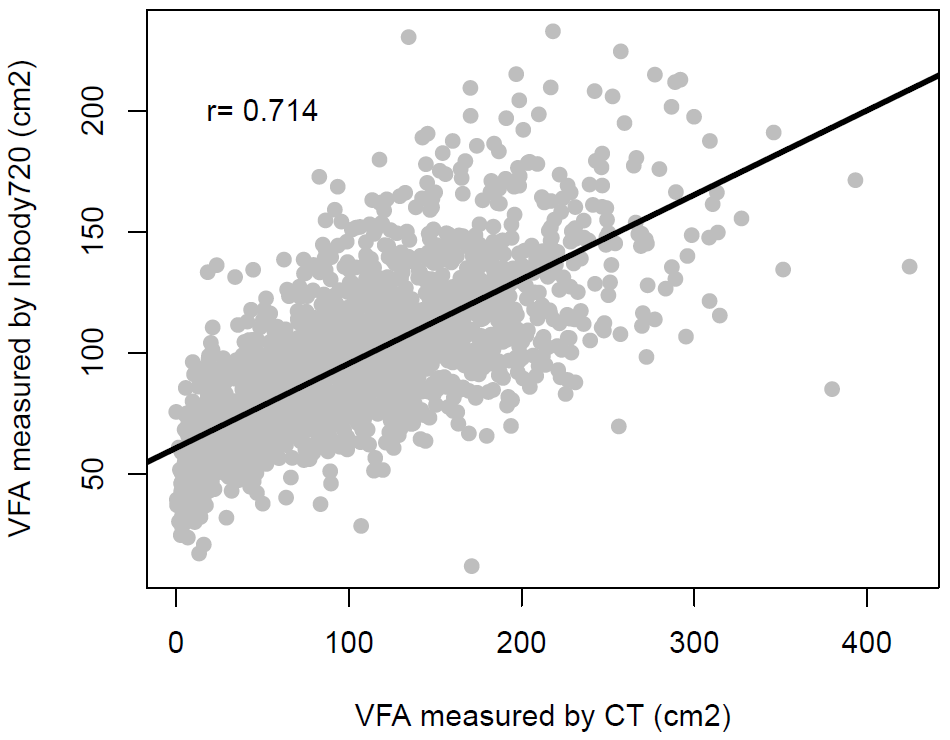

Supplement: Supplementary file 1 — Supplementary Figures. [file 41598_2023_30833_MOESM1_ESM.docx]
